# An Economic Evaluation of the Impact of Using Rapport-Based Interviewing Approaches With Child Sexual Abuse Suspects

**DOI:** 10.3389/fpsyg.2021.778970

**Published:** 2021-12-09

**Authors:** Susan Giles, Laurence Alison, Paul Christiansen, Michael Humann, Emily Alison, Ricardo Tejeiro

**Affiliations:** Department of Psychology, University of Liverpool, Liverpool, United Kingdom

**Keywords:** child sexual abuse, investigative interviewing, rapport, ORBIT, motivational interviewing, economic evaluation

## Abstract

Two studies examined whether rapport-based interviewing with child sexual abuse (CSA) suspects provides greater interview yield that could result in overall cost-savings to the investigation. First, multi-level modelling was applied to 35 naturalistic CSA suspect interviews to establish whether rapport-based interviewing techniques increase “yield” – defined as information of investigative value. The Observing Rapport Based Interviewing Technique (ORBIT coding manual was used to code interviews; it includes an assessment of both interpersonal adaptive and maladaptive rapport-based interviewer engagement as well as motivational interviewing (MI) strategies. The impact of these two strands (interpersonal and MI) on extracting information of investigative value (including strengthening a case for court and safeguarding) were examined. Adaptive interpersonal strategies increased case strengthening and safeguarding yield, with motivational interviewing having the largest impact on safeguarding yield. Both strategies increase the likelihood of gaining additional types of economic yield. Maladaptive interviewer strategies reduced case strengthening and different types of economic yield. In study two, literature-based economic estimates were applied to establish the potential cost benefits from following national ORBIT rapport training. Further training in adaptive and motivational interviewing could contribute cost savings between £19 and £78 million (annual unit costs) increasing to £238–£972 million (lifetime costs) for online CSA across England and Wales; and £157–£639 million (annual unit costs) increasing to £2–£8 billion (lifetime costs) for all CSA. Failure to commit training resource to this, or an alternative strategy, could mean the cost burden attributable to maladaptive interviewing (between £1 and £6 million for online CSA and £12 and £48 million for all CSA) is not successfully averted.

## Introduction

Despite low levels of disclosure and conviction rates, estimated at 12 and 17%, respectively (Children’s Commissioner for England, 2015), the number of reported cases of child sexual abuse (CSA) creates demanding workloads for policing bodies. Police in England and Wales recorded 73,260 sexual offences against children in the year ending March 2019 (27% rape offences and 12% with an online element; National Society for the Prevention of Cruelty to Children [NSPCC], 2020). This is equivalent to one sexual offence against a child recorded every 7 min, one child rape recorded every 30 min, and an internet offence against a child recorded every hour. Historical child sexual abuse (e.g., Hughes and Jonas, 2015) and CSA with an online element (Mitchell et al., 2010; Wolak et al., 2011; National Society for the Prevention of Cruelty to Children [NSPCC], 2020) have also increased. There are additional concerns that the internet has generated new forms of CSA offending with easy access to IIOC, children and other offenders online reinforcing deviant sexual interests (Kloess et al., 2014). Middleton (2009) estimated that one-third of CSA included an online element. In their meta-analysis Seto et al. (2011), found up to 55% of IIOC users admitted to a contact sexual offence, while Long et al. (2013) found 87% of dual offenders engaged in grooming behaviours both on and offline, and one fifth of IIOC viewers had produced IIOC (via webcams/grooming children to take photographs of themselves). Findings such as these prompted Christie (2018) to argue that IIOC offences, online grooming and offline CSA be treated as one offence type.

The National Crime Agency (2020) estimate there are now 300,000 individuals in the United Kingdom who pose a sexual threat to children, either through contact abuse or online. This requires a huge investment of time and resources to tackle the problems. Giles and Alison (2021) calculated that the Child Exploitation and Online Protection Command’s estimated national pool of 50,000 IIOC suspects (CEOP, 2013) could have already contributed an economic burden of £97–£445 million (incident costs) increasing to £1.2–£5.4bn (lifetime costs for services and victims) through historical contact CSA. If left unattended by targetted police action, historical victims would not be safeguarded and risk re-victimisation, and future contact offences could contribute a further burden of £16–£18.6 m (incident costs; £198–£227 m lifetime costs). Designated a national threat by the NCA in 2015, the scale of the problem now requires the development of evidence-led, cost-effective investigative techniques.

### Rapport-Based Investigative Interviewing With Child Sexual Abuse Suspects

Enhancing effective CSA suspect interviews should help improve outcomes. Suspect interviews represent a critical moment for investigators; to gather evidence against suspects that builds and strengthens cases and crucially, helps achieve safeguarding goals. Alison and colleagues argued that rapport-based strategies are preferable to high pressure interrogation techniques. Many studies have demonstrated the benefits of the Observing Rapport Based Interviewing Technique (ORBIT) with terrorist suspects and online child sexual abuse suspects (e.g., Alison et al., 2013; Christiansen et al., 2018; Surmon-Böhr et al., 2020). Indeed, surveys with prisoners confirm that interviewees are more responsive to rapport building and non-adversarial strategies (Kebbell et al., 2006, 2010; Cleary and Bull, 2019).

Responsiveness has been defined by Alison and colleagues as, first, the reduction in counter interrogation strategies and disengagement (e.g., aggression, complete silence or “no comment”) and, second, increased yield (information of evidential value). The ORBIT framework consists of three elements. The first two are independent sets of measures: one based on Motivational Interviewing skills (MI; Miller and Rollnick, 2009, 2012) and the other on the Interpersonal Behaviour Circle (IBC; Leary, 1957). In their examination of the ORBIT model, they have found that adaptive interpersonal strategies (which include adaptive means to handle difficult suspect behaviour), coupled with aspects of motivational interviewing (honesty, authenticity, and empathy) increased yield. In contrast, maladaptive interpersonal strategies (in which the interviewer exacerbates aggression of increases a commitment to silence or no comments) and behaviours antithetical to MI (dishonesty, leaking judgement, and indifference) reduces yield. This reflects the autonomy afforded to the suspect, allowing them to engage with the evidence and topics presented to them during interviews, without undue pressure or manipulation. The goal is to recognise the situation the suspect is in, consider the implications and draw out their feelings and beliefs.

### Economic Yield Linked to Suspect Interviews

Adherence to rapport-based interviewing should have numerous benefits. Firstly, revealed information might present opportunities to safeguard victims, including undisclosed victims, offences, and suspects, thereby reducing the considerable economic costs associated with sexual victimisation (Giles and Alison, 2021). Second, it would reduce workload as long delays with digital forensics to identify additional victims (e.g., Gallagher et al., 2006; Christie et al., 2015; Christie, 2018) may be avoided through it producing targetted intelligence. Third, targetted intelligence might also lead to additional corroborating evidence.

Corroborating evidence plays a key role in successful legal outcomes (e.g., Alonzo-Proulx and Cyr, 2017). Offline CSA often takes place in private with few eyewitnesses, with less physical evidence so investigators are often reliant on children’s testimony (Walsh et al., 2010). Along with problems associated with children’s accounts (e.g., developmental stage, ability to verbalise, memory, impact of grooming, shame, and self-blame) there are often further complications owing to pre-existing relationships with suspects meaning victims are reluctant to disclose or cooperate (Gekoski et al., 2016). With the increase in online offences there is the potential for further corroborating evidence (digital evidence or additional victims who can support the victim’s testimony), and rapport-based interviewing may help generate such detail and subsequently increase guilty pleas.

Walsh et al. (2010) found cases with an increased quantity of different types of supporting evidence were more likely to be convicted and to enter a guilty plea. A guilty plea means that victims do not need to appear in court, which can help to alleviate the associated detrimental impact on victims (e.g., Parsons and Bergin, 2010; Quas and Goodman, 2012; Gekoski et al., 2016; Joleby et al., 2020). Whilst this impact is difficult to monetise, if ORBIT can increase both the quality and quantity of evidence, it may reduce the amount of child victims required to appear in court.

Examples of interview yield that could be more directly monetised include information about specific locations and devices (e.g., passwords), information about specific movements of the suspect and other suspects, knowledge of other suspects, plans and devices, culpability for other offences and knowledge of other victims. Procuring this information would reduce resource requirements elsewhere (data analytics, digital forensic, house to house, victim identification, and identification of networks), facilitate safeguarding (disclosed and undisclosed victims), and help to strengthen cases ultimately improving legal outcomes (charging decisions, prosecutions, and convictions).

### Evaluation of Investigative Techniques

To date, there has been no economic evaluation of suspect interviewing approaches. There have, however, been evaluations that demonstrate the efficacy of other police actions. For example, multi-agency teams improve case outcomes following child abuse; specifically, child abuse assessment centres (Joa and Edelson, 2004) and child advocacy centres (Nwogu et al., 2015; Herbert and Bromfield, 2016, 2019a,b; Bracewell, 2018; Herbert et al., 2018). Rumney et al. (2020) also considered the case outcome benefits associated with police specialist rape investigation units, whilst Cloutier (2020) considered the use of specialised courts for sexual crimes. A number of studies have tested the efficacy of medical forensic examinations in helping to improve case outcomes following sexual violence (e.g., Menaker et al., 2017; Kjaerulff et al., 2019; La Harpe et al., 2019).

Limited research has been conducted to examine the investigative, legal and victim outcomes of investigative interviews. The most directly relevant to ORBIT is Kebbell et al. (2006), which reported that sex suspects who confessed to offences, perceived their interviewers to have been more ethical and to have displayed more humanity than those who denied offences. Moreover, the use of suspect interviews improves case outcomes (Kelley, 2008), evidence-based interview techniques with children increase charging rates (Pipe et al., 2013), and voice stress analysis elicits disclosures of undetected offences amongst offenders (Stathis and Marinakis, 2020). However, Sellers and Kebbell (2011) found interviewers’ disclosure of evidence against sexual abuse suspects did not impact decisions to confess. Pichler et al. (2020) reported that interview quality with child interviewees (open-ended questions, compliance with best practice, and evidential categories sought) was not associated with trial outcomes when controlling for number of victims and corroborative evidence. The strongest predictor of conviction was number of victims.

The purpose of the current research is to conduct an evaluation of naturalistic CSA suspect interviews to explore the extent to which adherence to rapport-based methods (specifically the ORBIT framework’s adaptive interpersonal behaviour coupled with motivational interviewing) elicit more relevant cost-saving information from suspects. Study 1 tests the extent to which rapport-based interviewing (increased adaptive, and reduced maladaptive, interviewing along with motivational interviewing) leads to suspects revealing increased evidential yield. In study 2, we draw on a systematic review strategy to establish the various cost estimates that could be associated with case strengthening and safeguarding economic yield. We then estimate the potential impact that national ORBIT training could have, in terms of cost savings generated from improving the use of rapport-based approaches along with outlining cost burdens generated from failure to execute rapport-based interviewing effectively. It is important to note that this is not a cost benefit analysis. This paper establishes the potential economic benefit, but in making any decisions on whether to implement the approach, further consideration should be given to the costs of doing so, as well as the risks.

## Materials and Methods

### Study 1 Multi-Level Modelling of Naturalistic Interviews

#### Participants

The case files for 25 CSA suspects were drawn upon for this research. Independent coders analysed 35 individual (as some cases contained multiple interviews) interviews across 44 h, broken down into 176 15-min segments. While the interviewers were not trained in ORBIT, they represented a sample of experienced officers (10+ years in specialist area, with at least PIP 2 or Tier 3 equivalent training) demonstrating a range of adaptive and maladaptive methods. The cases were selected to represent a diverse range of suspects and challenges presented during interview, which included disengaged or no-comment suspects, others denying knowledge of most evidence presented to them, and some that confessed and provided full accounts. They include both contact and IIOC only suspects, female and male suspects, multiple and single offender cases, international and United Kingdom based, as well as a diverse range of victims (e.g., contact abuse of children and teenagers, repeat image offences, online grooming, payment for live streaming content). Cases covered a range of forces throughout the United Kingdom.

#### Materials

Two experienced ORBIT coders, worked independently to code each 15 min interview segment according to an ORBIT coding manual. The manual developed by Alison and Alison (2012) was used to code the behaviour of the participants in the interview. Interviewer behaviour was coded into three elements measuring the following: (i) Global Motivational Interviewing Scores (GMIS), (ii) Motivational Interviewing of Detainees: Assessment of Skills (MIDAS), and (iii) Interpersonal Behaviour Circle: Adaptive and Maladaptive – Interviewer (IBC-I). Suspect behaviour was coded into two variables by measuring: (i) Interpersonal Behaviour Circle: Adaptive and Maladaptive – Detainee (IBC-D), and (ii) Interview Yield Assessment (IYA). Further details can be found in Alison et al. (2013). Uniquely for this study and the CSAE offender sample, the coding framework also included components relating to transition points, denial strategies (adapted from Sykes and Matza, 1957), and economic evaluation measures.

The first author worked with the coders to identify types of economic yield. Eight variables of economic yield were combined to form a variable, **case strengthening** – passwords and pin codes, along with evidence of involvement, usernames on social media, info on victim devices, presence of digital evidence, info on areas/movements, knowledge of other undisclosed devices, and knowledge of other significant people. Six variables of economic yield were combined to form a variable, **safeguarding**; usernames of associates, information about associate devices, knowledge of other undisclosed plans, other suspects, culpability for other offences, knowledge of other victims.

We did not have access to legal outcomes for individuals within this data set.

Cohen’s Kappa (k) was used to measure inter-coder agreement. The analysis indicated moderate to almost perfect agreement between coders with five instances of fair agreement between coders.

#### Analysis

Two binary dependent variables were created for the analysis. These were (1) **“Case strength,”** a score of one was given if in that measurement unit information was given on any of eight case strengthening variables, and (2) **“Safeguarding,”** a score of one was given if in that measurement unit information was given on any of the six safeguarding variables. A third continuous variable ‘Total economic yield’ was the summed score of all “case strength” and “safeguarding” variables elicited from suspects (up to a maximum of 14).

As the data consisted of multiple measures nested within the same suspects, this necessitated the use of multi-level general linear modelling (as independence of errors would be violated). We ran a variance components model with “suspect” as a random intercept and produced an intraclass correlation coefficient (ICC) to ascertain the percentage of variance in each outcome attributable to “suspect.” The variance components model was also compared to a single level model, to ensure fit of the former was superior [assessed with the *X*^2^ difference between models and the Akaike information criterion (AIC)].

We than ran a series of multi-level logistic regression models with a logit link and a Nelder–Mead optimiser for case strength and outcomes. For “total economic yield” multi-level Poisson regression with a log link was utilised. Regression coefficients along with odds ratio’s (or Relative risk ratio’s in the case of the Poisson model) and their 95% confidence intervals were reported. For each outcome four separate models were tested: (1) Overall adaptive, maladaptive and MI skills (2) Each of the global MI skills as the predictors, (3) Each aspect of adaptive interviewing as predictors and, (4) Each aspect of maladaptive interviewing as predictors.

### Study 2 Economic Modelling of Rapport-Based Interviewing

A rapid evidence assessment (see Varker et al., 2015), was undertaken to develop an economic framework to apply to investigative interviews. The aim was to summarise what is known about the cost of CSA investigations and average victim costs for CSA. The review of material followed in the economic tradition; producing a systematic literature review to provide an overview of available evidence from which we make critical decisions about figures in cost estimates.

First, we drew on the systematic review produced by Giles and Alison (2021). These authors undertook a systematic literature review in August 2020, searching for relevant material published 2000–2020, that met search terms (cost AND victim AND sex* crime OR rape OR child sex* abuse OR indecent image* OR internet sex* offend* OR online sex* offend OR contact sex offend OR groom* OR chat room off* or solicitation off* OR molest* OR pedo* OR paedo*) and could be found utilising six databases (Cochrane library, PsycINFO, Scopus, Web of Science, NCJRS, and PubMed). This was focussed on average victim costs for CSA. To expand upon this and identify resources examining the cost of CSA investigations a PICO framework was developed to establish any economic costs (outcomes) associated with police (population) action (intervention) during CSA investigations along with broader outcomes that could potentially be monetised (e.g., outcomes, including impacts on offending). Economic evaluation research would also be considered (control).

#### Search Strategy

Studies would be included if they included economic costs associated with CSA investigations, provided an economic assessment of police actions during CSA investigations, or demonstrated the cost effectiveness of such. Search terms were developed from the PICO (“police*” or “law enforcement”) AND (“invest*” OR “interview” OR “enquiry” OR “inquiry” OR “response” OR “evidence” OR “analysis” OR “case manag”) AND (“cost” OR “price” OR “value” OR “economic” OR “investment” OR “resources”) AND (“sex* crime” OR “sex* offender” OR “sex* offender victim” OR “child sex* abuse” OR “child porn*” OR “indecent image* of children” OR “IIOC” or “internet sex* offend*” OR “online sex* offend” OR “contact sex offend” OR “groom*” OR “chat room off*” or “solicitation off*” OR “molest*” OR “pedo*” OR “paedo*” OR “hebro”). The search covered the period from 2000 to 2020 and was not limited by language or publication type, or with respect to whether they were published or unpublished. Seven databases were searched in total: PsycINFO, Scopus, WoS, NCJRS, PubMed, EconLit, and repec. A request for relevant police literature was also disseminated by national policing leads.

Following initial searches, there followed de-duplication, review of study titles, application of screening procedures, and compilation of target lists for further review. Application of search terms resulted in 4,052 items. These were screened by the first author, such that items were excluded based on duplication, or on the basis that the full text did not inform the research question. A short list of 182 items were reviewed. Shortlisted material included a range of police interventions in child sexual abuse and sexual violence cases, including forensic advisors in court and multidisciplinary teams, but the majority were excluded on the basis that economic data was not presented.

#### Cost of Child Sexual Abuse Investigations

Three studies were retained that provided detailed accounts of the economic costs associated with CSA investigations (Christie et al., 2015; Christie, 2018; Heeks et al., 2018).

Costs provided by the Home Office (Heeks et al., 2018) are drawn upon nationally to help estimate the “disease burden” generated from crime. However, it is important to note that the costs provided are not unit costs to police for each crime recorded. The Home Office uses all crimes (rather than police recorded crimes) to estimate the unit cost for each crime. As such, the unit costs for each rape and other sexual offence will be higher. The total cost to the police by crime type (e.g., rape) is divided by the estimated total number of crimes (including those not reported to the police).

Christie et al. (2015) and Christie (2018) worked with United Kingdom policing partners to scope out the potential volume of CSA and relatedly, approximate costings for the police response across the United Kingdom. Both reports provide a detailed breakdown of potential costs throughout the life cycle of a typical online and offline CSA investigation that could be utilised for subsequent economic analysis. This provides a framework to be used in future research examining the benefits of police investments. As such, we draw upon the detailed costs provided by Christie et al. (2015) and Christie (2018) to inform our economic evaluation.

#### Average Child Sexual Abuse Victim Costs

Following a systematic review methodology, Giles and Alison (2021) considered Saied-Tessier (2014); Heeks et al. (2018), and Letourneau et al. (2018) for inclusion in economic analysis given their focus on either child sexual abuse, the full range of costs included and/or focus on the United Kingdom. Saied-Tessier (2014) was further discounted from analysis as unit costs were not available across a range of measures. They argue for the use of Home Office figures (Heeks et al., 2018; United Kingdom) to calculate incident costs. An American study (Letourneau et al., 2018) was selected to estimate lifetime costs on victims and society.

#### Incident Costs

To provide a cost per incident, Heeks et al. (2018) calculate the unit cost of rape as £39,360 and other sexual offences as £6,520 in 2015/16. Figures increase to £43,214 and £7,158 in 2019 using Bank of England online “inflation” calculator with inflation averaged at 3.2% a year (Giles and Alison, 2021). These figures are drawn on in the economic proofs provided in study 2.

#### Lifetime Costs – Lower and Upper Bound Estimates

Letourneau et al. (2018) provide average lifetime costs for victims of non-fatal child sexual abuse in the United States in 2015. Drawing upon a wider range of measures than Heeks et al. (2018) such as education costs, they also draw on the available child sexual abuse literature to establish lifetime effects on health, criminal offending, suicide death and QALY losses. They estimate that the lifetime costs for female victims of non-fatal child sexual abuse is $282,734. Applying HM Revenue & Customs Exchange Rates (2020) $282,734 was calculated as £176,850 in 2015, this figure increases to £197,535 in 2019 using Bank of England online “inflation” calculator with inflation averaged at 2.8% a year. This is used as a lower bound lifetime cost in the economic proofs provided in study 2.

Separately, Letourneau et al. (2018) estimate quality of life losses as $41,001 for female victims and $38,904 for male victims. Upper bound estimates could therefore include quality of life losses (adjusted to $40,477 to account for 75% of victims being female, see Giles and Alison, 2021). This was calculated as £25,318 in 2015 and increased to £28,279 in 2019 (using the same method). As such, the upper bound estimate of lifetime cost used in study 2 is £225,814.

Table 1 provides a detailed breakdown of costs that inform our economic framework, including our rationale for lower and upper bound estimates.

**TABLE 1:** Economic framework applied in the present research.

Economic yield	Rationale	Cost
Case strengthening – eliciting information contained within these eight variables could help officers to build stronger cases using corroborating evidence. Most of these details draw on the types of evidence outlined by Walsh et al. (2010) and the figures provided by Christie et al. (2015; Christie, 2018)		
**Password data**	Cost savings in terms of reduced time needed by digital forensic teams	
*Lower bound estimate*	*Assuming that a password saves the digital forensics team one hour of work Failure to elicit a password would constitute £16 cost burden* *	**£16 per case** (1 h × civilian staff grade)
*Upper bound estimate*	*Assuming that a password expedites subsequent analysis, an assumption of 10% reduction in total costs*	**£101 per case**
**Targeted witnesses and CCTV**	Cost of enquiries and to view footage, involving 2 detectives working 8 h each enquiry. Detectives costed at £19.71p/h, total cost £315 per case	
*Lower bound estimate*	*Gaining targeted intelligence from suspects could reduce this time by 10%*	**£31.50 per case**
*Upper bound estimate*	*Gaining targeted intelligence from suspects could reduce this time by 50%*	**£157.50 per case**
**Locations and interviews**	Estimated cost of £532 per case for houses or other locations attended along with typical number of people interviewed per victim	
*Lower bound estimate*	*Gaining targeted intelligence from suspects could reduce this time by 10%*	**£53.20 per case**
*Upper bound estimate*	*Gaining targeted intelligence could negate need for 1 location and 1 interview (6 h per case)*	**£118.26 per case**
**Reduced length of investigation**	Based on the assumption that 50% of cases proceed to prosecution and average wait time is 9 months, officers needing to maintain regular monthly visits are costed at £118.26 per month and 6 monthly child protection review meeting are costed at £79.85	
*Lower bound estimate*	Cases are processed within 8 months (1 month quicker) Reduction in monthly home visits	**£118.26 per case**
*Upper bound estimate*	Cases are processed within 6 months (3 months quicker) Reduction in monthly home visits 6 monthly child protection review meeting not needed	**£354.78 £78.85**
**Guilty plea negates court costs**	Based on the assumption that 50% of cases proceed to prosecution, considering police time for court appearances across online and offline offences and witness care in offline offences, the average court costs are estimated at £1,340.50	
*Lower bound estimate*	Increase in case strength economic yield, linked to rapport-based interviewing, contributes to 1% increase in guilty pleas	**£1,340.5 cost saving (1% cases)**
*Upper bound estimate*	Contributes to a 10% increase in guilty pleas	**£1,340.5 cost saving (10% cases)**
Safeguarding – Eliciting information contained in these six variables could help officers identify and safeguard additional victims, along with victims of undetected offenders. Drawing upon Home Office incident costs (Heeks et al., 2018). Giles and Alison (2021) provide a lower bound estimate for sexual offences, calculating incident costs as £43,214 for 25% offences involving rape, and £7,158 for 75% of cases involving non-penetrative sexual assault. This is in line with Office of Office of National Statistics [ONS] (2020) figures (27% of cases involved rape). Lifetime costs are adapted from a prevalence study conducted by Letourneau et al. (2018; United States). Lifetime costs are estimated at £197,535 (lower bound) increasing to £225,814 (including QALY; upper bound)		
**Additional victims safeguarded**	In this study, we adopt 12 and 55% as lower and upper bound risk estimates for contact sexual offending (drawing upon Seto et al., 2011; as in Giles and Alison, 2021) but we make an additional assumptions here. As we know some of these individuals are contact offenders [e.g. 27% rape offences, Office of Office of National Statistics [ONS], 2020] we instead assume that 12 and 55% of offenders have one additional victim that is potentially safeguarded and so the cost of their victimisation is successfully averted. These cases would theoretically require subsequent investigation and so it would be inappropriate to assume police cost savings, as such the cost of a police investigation provided by Heeks et al. (2018) is subtracted from the above mentioned unit costs	
*Unit costs lower bound estimate*	Assume 12% offenders have additional victim, with 25% rapes and 75% sexual assaults	**£36,854 per rape £6,588 per sexual assault (12% cases)**
*Unit costs upper bound estimate*	Assume 55% offenders have additional victim, with 25% rapes and 75% sexual assaults	**£36, 854 per rape £6,588 per sexual assault (55% cases)**
*Lifetime costs lower bound estimate*	Assume 12% offenders have additional victim	**£191,175**
*Lifetime costs upper bound estimate*	Assume 55% offenders have additional victim	**£219,454 (with QALY)**
**Reduction in recidivism**	Again, following the methodology established in Giles and Alison (2021) we draw upon Seto et al. (2011) reoffending rates to provide a lower bound estimate and Wakeling et al. (2011) dual offending recidivism rates to provide an upper bound estimate. At one potential victim averted per offender this is considered to be a conservative strategy. Block et al. (2013) for example, drew on Abel’s (1985) estimate of 117 victims for non-familial CSA offenders to estimate that a 5% reduction in offending following conviction could lead to 6 fewer children being subsequently offended again. Here we estimate only one child	
*Unit costs lower bound estimate*	2% offenders with additional contact victim and 3.4% additional internet offence, cost averted using Heeks et al. (2018) adapted figure, 25% offences at £36,854 (rape) and 75% at £6,588 (non-penetrative sexual assault)	
*Unit costs upper bound estimate*	2.3% offenders with additional contact victim and 4.6% additional internet offence, cost averted using Heeks et al. (2018) adapted figure, 25% offences at £36,854 (rape) and 75% at £6,588 (non-penetrative sexual assault)	
*Lifetime costs lower bound estimate*	2% offenders with additional contact victim and 3.4% additional internet offence, cost averted using Letourneau et al. (2018) adapted figure, £191,175 (£219,454 including QALY)	
*Lifetime costs upper bound estimate*	2.3% offenders with additional contact victim and 4.6% additional internet offence, cost averted using Letourneau et al. (2018) adapted figure, £191,175 (£219,454 including QALY)	

*This rationale is carried throughout the analysis, failure to elicit such information is identified as the same amount of money but as a cost burden.

#### Analysis

The economic framework is applied to national crime reports to establish the potential impact we might expect from ORBIT training, moving the interviewer workforce toward predominant use of adaptive and motivational interviewing. This approach also helped to establish the potential cost burden associated with predominant use of maladaptive interviewing. The economic estimates are applied to the most recently published crime figures for online CSA [*n* = 8,807; National Society for the Prevention of Cruelty to Children [NSPCC], 2020] and all CSA recorded by police forces in England and Wales [*n* = 73,260; Office of Office of National Statistics [ONS], 2020].

## Results: Study 1

### Case Strengthening

Case strength–is the presence of *any* of the case strength variables (91) vs. absence (81). Case strength is coded as 0/1 with the latter indicting presence.

#### Variance Components

The measurement within case model (AIC = 231.28) was a better fit compared to single level model [AIC = 238.86; *X*^2^(1) = 10.581, *p* = 0.001, ICC = 0.21]. This indicated a multi-level model is required.

#### Multi-Level Models

These tables show a one unit increase in adaptive interviewing strategies produce a 35% increase in the likelihood of gaining economic yield that would strengthen cases (Table 2). Notably the use of adaptive co-operative strategies appears most effective with a one unit increase here increasing the likelihood of gaining case strength economic yield by 55% (Table 3).

**TABLE 2:** Adaptive, maladaptive, and MI (case strength).

	*B*	SE	*P*	OR	95% CI
Maladaptive	−0.243	0.114	0.033	0.78	0.63–0.98
Adaptive	0.299	0.102	0.003	1.35	1.11–1.65
MI	−0.037	0.145	0.412	0.96	0.88–1.05

**TABLE 3:** Adaptive interviewing (case strength).

	*B*	SE	*P*	OR	95% CI
Authoritative	0.245	0.186	0.188	1.27	0.89–1.84
Cooperative	0.438	0.201	0.030	1.55	1.04–2.30
Passive	0.294	0.178	0.095	1.34	0.95–1.89
Confrontational	−0.172	0.145	0.235	0.84	0.63–1.12

One unit increase in maladaptive interviewing reduces the likelihood of gaining case strength economic yield by 22% (Table 2). This is particularly related to the use of passive maladaptive strategies; one unit increase here decreases the likelihood of gaining case strength economic yield by 48% (Table 4). Motivational interviewing does not appear to be related to case strength economic yield (Table 5).

**TABLE 4:** Maladaptive interviewing (case strength).

	*B*	SE	*P*	OR	95% CI
Authoritative	−0.134	0.339	0.694	0.87	0.45–1.70
Cooperative	0.474	0.482	0.326	1.61	0.62–4.13
Passive	−0.648	0.234	0.006	0.52	0.33–0.83
Confrontational	−0.360	0.249	0.148	0.68	0.43–1.14

**TABLE 5:** Motivational interviewing (case strength).

	*B*	SE	*P*	OR	95% CI
Acceptance	0.494	0.299	0.098	1.64	0.91–2.94
Adaptation	−0.037	0.255	0.884	0.96	0.58–1.59
Autonomy	0.206	0.286	0.471	1.23	0.70–2.15
Evocation	0.094	0.312	0.767	1.10	0.60–2.05
Empathy	−0.270	0.329	0.412	0.076	0.49–1.46

### Safeguarding

Safeguard is the presence of *any* of the safeguarding variables (36) vs. absence (136). Safeguard is coded as 0/1 with the latter indicating presence.

#### Variance Components

Measurement within case model (AIC = 174.09 was a better fit compared to single level model [AIC = 178.48; *X*^2^(1) = 6.395, *p* = 0.011, ICC = 0.29]. Although the difference was significant the AIC did not indicate a substantial improvement from the single level to multilevel model (AIC difference <5), and wide large CIs generated. We repeated the safeguard analysis using a single level, Penalised likelihood regression analysis (logistic regression using Jeffery’s invariant prior).

A one unit increase in motivational interviewing strategies produced a 12% increase in the likelihood of gaining safeguarding economic yield (Table 6). This is particularly related to the use of evocation; one unit increase here doubles (222%) the likelihood of gaining safeguarding economic yield (Table 7). Adaptive interviewing approaches also play a part. Whilst overall adaptive interviewing was not related to increased safeguarding economic yield, it is notable that a one unit increase in passive adaptive interviewing is associated with a 57% increase in the likelihood of gaining safeguarding economic yield (Table 8). Maladaptive interviewing does not appear to be related to safeguarding economic yield (Table 9).

**TABLE 6:** Adaptive, maladaptive, and motivational interviewing (safeguard).

	*B*	SE	*p*	OR	95% CI
Maladaptive	0.020	0.115	0.757	1.02	0.81–1.27
Adaptive	0.030	0.098	0.86	1.03	0.85–1.25
MI	0.110	0.041	0.006	1.12	1.03–1.21

**TABLE 7:** Motivational interviewing (safeguard).

	*B*	SE	*p*	OR	95% CI
Acceptance	−0.043	0.309	0.888	0.96	0.52–1.74
Adaptation	0.059	0.261	0.819	1.06	0.64–1.77
Autonomy	−0.172	0.302	0.564	0.84	0.46–1.50
Evocation	0.785	0.345	0.017	2.22	1.15–4.44
Empathy	−0.039	0.318	0.902	0.96	0.52–1.80

**TABLE 8:** Adaptive interviewing (safeguard).

	*B*	SE	*p*	OR	95% CI
Authoritative	0.197	0.188	0.296	1.22	0.84–1.76
Cooperative	−0.161	0.209	0.615	0.85	0.55–1.26
Passive	0.454	0.182	0.009	1.57	1.12–2.28
Confrontational	−0.151	0.143	0.283	0.86	0.64–1.13

**TABLE 9:** Maladaptive interviewing (safeguard).

	*B*	SE	*p*	OR	95% CI
Authoritative	0.127	0.257	0.139	1.14	0.54–2.10
Cooperative	0.066	0.385	0.082	1.93	0.92–4.10
Passive	−0.339	0.241	0.142	0.71	0.43–1.11
Confrontational	−0.297	0.269	0.251	0.74	0.41–1.22

### Different Types of Economic Yield

Economic data is coded as information not given (0) or given (1) e.g., passwords, other offender names, etc., across 14 different categories. A total count of this information (#/14) was computed.

This count was then analysed with a series of multi-level Poisson regressions. Fit was compared against alternative models (zero inflated Poisson, negative binomial, and zero-inflated negative binomial) with the standard Poisson being the best fit as assessed with AIC values.

#### Variance Components

38.8% of variance in information produced was attributable to the case level, the remaining 61.2% to the unit of measurement. This suggests that the individual is not the most important factor in whether they gave this information or not (i.e., it’s not the case that some interviewees just talk and other do not at all, all showed variance in information given across the interview process). The dispersion for all three models was <1.06, meeting Poisson assumptions.

#### Global Motivational Interviewing Skills

Evocation had a significant effect on increasing the amount of information given Relative Risk Ratio (RRR) = 1.38, 95% CIs = 1.10–1.74, *p* = 0.006. This means that a unit increase in economic yield (one additional type of economic yield) is 38% higher when exposed to a 1 unit increase in evocation. There were no other significant MI skills.

#### Interviewer Adaptive

Adaptive cooperative interviewing significantly increased the amount of information given RRR = 1.21, 95% CIs = 1.03–1.44, *p* = 0.012 as did adaptive passive RRR = 1.23, 95% CIs = 1.07–1.40, *p* = 0.003. This means that a unit increase in economic yield (one additional type of economic yield) is 21% higher when exposed to a 1 unit increase in adaptive cooperative interviewing and 23% higher when exposed to a 1 unit increase in adaptive passive interviewing.

Notably adaptive confrontational had a negative association with information RRR = 0.87, 95% CIs = 0.77–0.98, *p* = 0.026. This means that a unit increase in economic yield (one additional type of economic yield) is 13% less likely when exposed to a 1 unit increase in adaptive confrontational interviewing.

#### Interviewer Maladaptive

Maladaptive passive interviewing had a significant negative effect on information given RRR = 0.74, 95% CIs = 0.61–0.91, *p* = 0.004, as did maladaptive confrontational RRR = 0.73, 95% CIs = 0.65–0.94, *p* = 0.017.

This is an interesting observation in respect to passive interviewing, done well (adaptive passive) increases the likelihood of number of different types of economic yield. Done badly (maladaptive passive) reduces the likelihood of number of different types of economic yield, here a 1 unit increase was associated with a 26% reduction in likelihood of gaining an additional type of economic yield. Equally, a 1 unit increase in maladaptive confrontational is associated with 27% reduction in likelihood of gaining an additional type of economic yield.

## Results: Study 2

### Case Strengthening

Adaptive co-operative strategies proved effective in improving the likelihood of gaining case strengthening economic yield. Moving the national workforce from none co-operative strategies to predominant style of interaction (a three point unit increase) could be associated with an 165% increase in likelihood of gaining case strength economic yield. Whilst the initial base line of economic yield was low, this analysis has demonstrated potential benefits associated with increase in adaptive co-operative strategies in a naturalistic setting (i.e., these interviewers were not ORBIT trained). In Table 10 we apply the economic framework provided in Table 1 to project the potential cost savings that could be associated with ORBIT training on a national level, responding to annual reports of online CSA and all CSA in England and Wales.

**TABLE 10:** Projected cost savings attributable to predominant use of adaptive cooperative interviewing.

Cost	*n* = 8,807 online CSA		*n* = 73,260 all CSA	
	Lower bound	Upper bound	Lower bound	Upper bound
1. Passwords	£140,912	£889,507	£1,172,160	£7,399,260
2. Better targeted enquiries (witness/CCTV)	£227,420.50	£1,387,102.50	£2,307,690.00	11,538,450.00
3. Better targeted enquires (other)	£468,532.40	£1,041,515.83	£3,897,432.00	£8,663,727.60
4. Less time awaiting trial	£520,817.04	£1,909,706.52	£4,331,863.80	£15,883,866.90
5. Court costs	£58,982.00	£589,820.00	£491,293.25	£4,910,252
Total	£1,416,664	£5,817,652	£12,200,439	48,395,557

Taken together, predominant use of adaptive co-operative strategies could reduce police time (passwords, targetted case building, shorter investigations, and reduced court costs through guilty pleas) contributing annual cost savings for police forces across England and Wales; between £1.4 and £5.8 million for online CSA and £12.2 and £48.4 million all CSA.

Using the same logic applied to passive maladaptive interviewing (48% reduced likelihood of case strength economic yield), predominant use of passive maladaptive adaptive interviewing could reduce the likelihood of gaining case strength economic yield by 144%. Failure to commit resources to reduce the use of passive maladaptive interviewing could generate a cost burden within the same magnitude as the cost saving established for adaptive co-operative strategies (between £1.4 and £5.8 million for online CSA and £12.2 and £48.4 million all CSA). As cost effective as adaptive interviewing is, maladaptive interviewing could prove equally costly.

### Safeguarding

Evocation and passive adaptive interviewing proved effective in improving the likelihood of gaining safeguarding economic yield. Moving the national workforce from no evocation to predominant style of interaction (a three-point unit increase) could mean that interviewers are up to six times more likely to elicit safeguarding economic yield from interviewees. Moving toward predominant use of passive adaptive interviewing would be associated with 171% increased likelihood of gaining safeguarding yield. The initial baseline of safeguarding economic yield was higher than case strengthening, and this analysis has demonstrated further potential benefits associated with increase in evocation and passive adaptive strategies in a naturalistic setting (i.e., these interviewers were not ORBIT trained). In Tables 11, 12 we apply the economic framework provided in Table 1 to project the potential cost savings that could be associated with ORBIT training on a national level, responding to annual reports of online CSA and all CSA in England and Wales.

**TABLE 11:** Additional victims safeguarded – estimated at one victim per offender.

	Offending prevalence	Contact offenders (*n*)	Incident costs^1^	Lifetime costs^2^	Lifetime costs (with QALY)^3^
8,807 recorded online CSA	Lower bound: 12% (Seto et al., 2011)	1,057	£14,953,740	£202,071,975	£231,962,878
	Upper bound: 55% (Seto et al., 2011)	4,844	£68,564,398	£926,051,700	£1,063,035,176
73,260 all recorded CSA	Lower bound: 12% (Seto et al., 2011)	8,791	£124,439,776	£1,680,619,425	£1,929,220,114
	Upper bound: 55% (Seto et al., 2011)	40,293	£570,319,702	£7,703,014,275	£8,842,460,022

*^1^Estimate 25% offences at £36,854 (penetrative) and 75% at £6,588 (non-penetrative) in 2019 GBP.*

*^2^Estimated at £191,175 in 2019 GBP.*

*^3^Estimated at £219,454 in 2019 GBP.*

**TABLE 12:** Reductions in recidivism – estimated at one victim per offender.

		Reoffending prevalence	Recidivists (*n*)	Incident costs^1^	Lifetime costs^2^	Lifetime costs (with QALY)^3^
Reported online CSA	Future contact offending	Lower bound: 2% (Seto et al., 2011)	176	£2,846,272	£34,766,160	£39,743,264
		Upper bound: 2.3% (Wakeling et al., 2011)	203	£3,293,720	£40,099,605	£45,840,242
	Future internet offending	Lower bound: 3.4% (Seto et al., 2011)	299			
		Upper bound: 4.6% (Wakeling et al., 2011)	405			
All reported CSA	Future contact offending	Lower bound: 2% (Seto et al., 2011)	1,465	£22,386,546	£289,388,775	£330,817,510
		Upper bound: 2.3% (Wakeling et al., 2011)	1,685	£27,240,806	£332,846,475	£380,496,590
	Future internet offending	Lower bound: 3.4% (Seto et al., 2011)	2,491			
		Upper bound: 4.6% (Wakeling et al., 2011)	3,370			

*^1^Estimate 25% offences at £43,214 (penetrative) and 75% at £7,158 (non-penetrative) in 2019 GBP.*

*^2^Estimated at £197,535 in 2019 GBP.*

*^3^Estimated at £225,814 in 2019 GBP.*

Taken together, predominant use of passive adaptive interviewing and evocation could reduce potential victim harm (identification of other victims and reductions in reoffending) contributing cost savings for police forces across England and Wales; between £17.8 and £71.9 million for online CSA and £146.8–£597.6 million all CSA. These figures are based on unit costs. Cost savings, based on averted lifetime costs on victims increased to between £236.8 and £966.2 million for online CSA and £1.97-£8 billion all CSA (without QALY).

## Discussion

The scale of the CSA problem necessitates the development of evidence-led, cost-effective investigative techniques that facilitate legal outcomes and safeguarding goals. Faced with limited, and competing demand on, resources economic evaluation can help policy makers and law enforcement agencies decide where to allocate limited resources. The hypotheses were that rapport-based suspect interviewing generates economic yield that can (a) strengthen cases, and thus potentially save valuable police resources; and (b) help generate information about victims and other suspects, thus contributing to safeguarding goals.

Drawing on findings from study 1 and study 2 with respect to case strengthening, we found that adaptive co-operative strategies could potentially reduce police time (targetted case building, shorter investigations, and reduced court costs through guilty pleas) contributing annual cost savings for police forces. As cost effective as adaptive interviewing is, our analysis reveals that maladaptive interviewing is equally costly. The economic burden generated through increased use of passive maladaptive strategies was estimated as being equivalent to the estimated cost savings for cooperative adaptive interviewing.

In our second set of analyses, we found that predominant use of passive adaptive interviewing and evocation could reduce potential victim harm contributing cost savings across England and Wales; between £17,800 and £71,858 million for online CSA and £147 and £598 million all CSA. These figures are based on unit costs. Cost savings, based on averted lifetime costs on victims increased to between £237 and £966 million for online CSA and £2–£8 billion all CSA (without QALY). Evocation – an MI consistent technique directed at extracting thoughts feelings and beliefs appears to be a central mechanism here in encouraging suspect engagement and, subsequently, information of investigative value. Moving the work force toward consistent use of evocation could increase the likelihood of each trained officer gaining safeguarding information six-fold.

In summary, findings from our economic modelling suggest that the total cost savings emerging from adaptive interviewing and motivational interviewing (passwords, case strength, and safeguarding) could be between £19 and £78 million for online CSA using unit costs, this increases to £238–£972 million taking into account averted lifetime costs on victims. The estimated cost savings from adaptive interviewing and motivational interviewing (passwords, case strength and safeguarding) are estimated to be between £157 million and £639 million for all CSA using unit costs, this increases to £2–£8 billion taking into account averted lifetime costs on victims. The disadvantages of maladaptive interviewing could generate a cost burden on police forces across England and Wales, between £1,417 and £5,818 million for online CSA, and between £12,200 and £48,396 million for all CSA.

It is important that police forces recognise that the findings from this research (particularly study 1) are based on advantages of naturally occurring rapport-based strategies (along with disadvantages of naturally occurring maladaptive behaviours). This research does not provide a definitive evidence base for the full economic benefits of ORBIT. These interviewers had not been ORBIT trained. As demonstrated in the CT interviewing context (Alison, Plummer and Humann, under review), the anticipated benefits of an ORBIT trained workforce would be expected to be much higher. Even the limited number of rapport-based strategies that were observed conferred economic advantages.

A more detailed example is also useful for academics and practitioners to understand our use of odds ratios in study 2. We shall provide an example based on our findings around evocation. Findings from study 1 demonstrated that interviewers who used evocation (e.g., minimal expression of evocation) were twice more likely to elicit safeguarding yield than those interviewers in the category below them (e.g., no evocation observed). This increase in the likelihood of gaining safeguarding yield is linked to an officer moving one unit increase in their use of evocation (from no use to minimal use, minimal use to moderate use, moderate use to predominant use). Although not technically a linear prediction, the assumption is that the IRR has the same effect at each step. For study 2, we apply the finding of a two-fold increase per unit change to six-fold increase across a three unit change (moving the workforce from no use to predominant style of interaction). So if interviewers can be trained to move from not using evocation to using evocation consistently this *could* have *up to* a six fold increase in the likelihood of them eliciting safeguarding yield from suspects. This does not guarantee certainty as it is reliant on the baseline expression of evocation prior to training. However, it does suggests that officers encouraged to include even minimal expressions of evocation would be twice more likely to elicit safeguarding yield than officers who do not currently use evocation. Those who use evocation predominantly would be anticipated to be six times more likely to elicit safeguarding yield than those officers who do not use evocation at all. This is the anticipated improvement modelled from naturally occurring evocation and, as mentioned above, does not directly test the likelihood of gaining safeguarding yield following ORBIT training. The actual increase following ORBIT training may be different (confidence intervals must also be inspected). A note of caution must also be made about extrapolating these predictions. It may be that the biggest increase in odds emerges from moving from no use of evocation to minimal expression rather than from moderate expression to predominant expression. Further investigation would be needed to explore the exact effects of different levels of evocation.

Evocation, adaptive cooperative and adaptive passive interviewing were further found to increase the number of different types of economic yield. Conversely, adaptive confrontational, maladaptive passive, and maladaptive confrontational reduced the number of different types of economic yield. These results suggest that passive interviewing done well can increase yield, whilst done badly decreases yield. Confrontational interviewing reduces yield whether done in an adaptive or maladaptive way. Given findings from Walsh et al. (2010) that successful legal outcomes are dependent upon amount of corroborative evidence, these findings provide further evidence that rapport-based interviewing could have a direct impact on legal outcomes, potentially helping to reduce the poor rates of attrition and prosecution in CSA offences.

The evidence presented in this paper is promising, but on its own should not be used to justify investment in ORBIT. Further work is needed to establish a full cost benefit analysis. We are satisfied that our approach can be used to analyse future benefits. We have sought throughout to provide conservative figures so as to promote confidence in our approach. We have drawn on literature-based estimates, identified lower and upper bound estimates, used official crime reports, assumed one current and one future victim, employ recidivism rates based on apprehended offenders. In addition, we do not include the costs of averted internet offences. This is likely to include children who are sexually abused online, along with IIOC offences. As the literature on online CSA demonstrates, online CSA victims can be expected to experience similar levels of harm experienced by offline CSA victims (Whittle et al., 2013; Hamilton-Giachritsis et al., 2017). Further, the economic harm presented here does not consider impact of attending court for victims or family members, nor the wider impact on family members. These estimates might also be best seen as relating to adult offending as offences involving adolescents may not necessarily be recorded as CSA. Christie (2018) notes that 45% of offences are committed by adolescents. It is likely that costs emerging from adolescent offenders are considerable and that rapport-based interviewing would be equally advantageous with younger offenders.

Although conservative, there are limitations with the current study. First, we rely on single studies as the basis of estimates rather than meta-analyses. However, there are limited systematic reviews and meta-analyses available. Our analysis provides “back-of-the-envelope” calculations to demonstrate the potential cost saving that could emerge from estimated decreases in police time. This is a fairly established approach to demonstrate cost benefits (e.g., Wen et al., 2014) when trying to get a handle on potential costs. Whilst we recognise limitations with this approach, Christie et al. (2015) and Christie (2018) worked with partner forces to establish costs, adding validity to our approach. A sensible way forward would be to seek validation from policing partners for the estimates provided in this research. Second, we cannot rule out the role of other important variables or reverse causality, i.e., variables other than rapport-based interviewing led to economic yield, or that suspects willing to make admissions generated a more positive response from interviewers. Cases involving IIOC suspects may be more likely to lead to a guilty plea as there is a physical record of the abuse. It may also be that cases that suspects who assume strong evidence against them are more likely to make admissions during a suspect interview. To help address these potential problems, the ORBIT research team were clear to make sure where economic yield was revealed as a result of interviewers’ asking questions and where it was revealed by suspects’ own omission. We conducted economic analysis on economic yield that was revealed following asks. A further quality check would be to assess guilty pleas against the coding framework, i.e., is it the case that interviewers demonstrated rapport-based approaches with co-operative interviews who were likely to confess anyway, or did rapport-based interviewing generate yield and potential confession from recalcitrant interviewees? The coders noted that the use of rapport-based strategies was fairly limited in this naturalistic sample, but where present it was clear this had a positive effect in terms of gaining yield and economic yield. An alternative, beneficial approach, would be to conduct methodological triangulation on the existing data set using a qualitative approach to rule out the influence of additional factors and reverse causality.

There are a number of additional limitations with our cost estimates. First, some of our case strengthening estimates were based on the assumption that 50% of cases led to a charging decision and this may run the risk of inflating figures. Further work is needed here to establish whether 50% was a sensible metric or whether we should revise our figures with a smaller number (e.g., 17% as outlined by Children’s Commissioner, 2015). Second, our estimates assume that victims disclose their abuse and so use the services making up the bulk of tangible costs. However, as previously mentioned, as few as 12% of CSA victims might be expected to report offences to the police. Third, Heeks et al. (2018) do not specifically identify costs of sexual violence with child victims. As noted by Giles and Alison (2021) this has likely led to cost underestimation but further work examining child related costs would be clearly beneficial. Fourth, the evidence underlying assumptions in Letourneau et al. (2018) is United States centric. We attempted to interrogate the equivalent literature base in the United Kingdom, drawing upon databases routinely used within Health Economics as well as Psychology but no further references were identified. Fifth, some measures in Letourneau et al. (2018) are underdeveloped (e.g., educational impacts are measured using the typical costs of special education and this does not do justice to the profound educational impacts following sexual violence; Bolger, 2016). Sixth, Letourneau et al.’s (2018) estimates are based on victimisation at age 11 years. Further work is needed to explore variation in victim costs as a function of victim age (Fisher et al., 2017). Whilst our review did not reveal recidivism studies (beyond Elliott et al., 2019 which suggested higher recidivism rates than those employed here) future work might consider more recent advancements in recidivism studies (e.g., Hogan and Sribney, 2019; Coulter et al., 2021). Whilst problematic, we do not envisage these issues inflated costs. Rather, with further clarity, we would expect costs to increase.

This research has demonstrated the benefits of rapport-based strategies for eliciting economic yield. This knowledge can help interviewers appreciate and understand the potential benefits of rapport-based approaches and disadvantages associated with maladaptive approaches. The use of rapport-based approaches was low across the sample as a whole and interviewers did not routinely push for economic yield. In naturalistic settings, interviewers may be reliant on existing digital evidence to help support reactive interview strategies rather than being proactive in using interview strategies to help gain corroborative evidence. Interviewers would benefit from further knowledge and training about the role and value of rapport building in helping to push for and gain such information. Given the low levels of rapport-based skill in this naturalistic sample, it might also be useful to consider gaining the views of interviewers on rapport-based approaches (e.g., their concerns, any barriers they foresee in implementing such an approach). There may be reluctance to implement rapport-based strategies given the stigma of child sexual abuse offenders. Police officers, as well as police managers and the general public, may implicitly favour punishment and retribution in all phases of the criminal justice system, irrespective of the science and potential value of rapport-based approaches. This may present a barrier to the implementation of evidence-based practise. We might also consider more practical aspect, such as whether ORBIT takes longer to deliver and whether this is perceived as problematic for interviewers (see Brunel and Py, 2013). In future research, we might also consider whether or not ORBIT is effective in detecting false allegations of CSA and the potential economic implications emerging from this.

Drawing on evidence-based policing approaches, this research set out to establish whether rapport-based interviewing of CSA suspects represents an effective use of police resources. Multi-level modelling validated the potential mechanisms by which rapport-based interviewing increases economic yield. Economic modelling demonstrated the potential cost savings attributable to adaptive interviewing and cost burden associated with maladaptive interviewing. Evocation played a key role in eliciting safeguarding-related information from suspects. We anticipate that the costs presented here are conservative, as the analysis was conducted prior to any organised training programme for CSAE investigating officers to improve their use of rapport-based approaches when interviewing. With investment in training and improvements to interview approaches, the gains would be predicted to increase substantially.

## Data Availability Statement

The datasets presented in this article are not readily available because the datasets are subject to controlled access to police data. Requests to access the datasets should be directed to LA, alisonl@liverpool.ac.uk.

## Ethics Statement

The studies involving human participants were reviewed and approved by the University of Liverpool Central University Research Ethics Committee. Written informed consent for participation was not required for this study in accordance with the National Legislation and the Institutional Requirements.

## Author Contributions

SG designed the economic evaluation framework, undertook the systematic review described in study 2, and wrote the final manuscript. LA provided strategic oversight of the research, gained research funding for the project, contributed to manuscript writing, was one of the original developers of the ORBIT framework. PC produced the statistical models presented in study 1 and contributed to manuscript writing. MH provided project management and coded half of the interviews using the ORBIT framework. EA was the one of the original developers of the ORBIT framework and coded half of the interviews using the ORBIT framework. RT reviewed ORBIT coding and undertook inter-rater reliability analyses. All authors contributed to the article and approved the submitted version.

## Conflict of Interest

The authors declare that the research was conducted in the absence of any commercial or financial relationships that could be construed as a potential conflict of interest.

## Publisher’s Note

All claims expressed in this article are solely those of the authors and do not necessarily represent those of their affiliated organizations, or those of the publisher, the editors and the reviewers. Any product that may be evaluated in this article, or claim that may be made by its manufacturer, is not guaranteed or endorsed by the publisher.
